# Fast-track identification of CTX-M-extended-spectrum-β-lactamase- and carbapenemase-producing *Enterobacterales* in bloodstream infections: implications on the likelihood of deduction of antibiotic susceptibility in emergency and internal medicine departments

**DOI:** 10.1007/s10096-021-04192-8

**Published:** 2021-02-17

**Authors:** Matteo Boattini, Gabriele Bianco, Marco Iannaccone, Davide Ghibaudo, André Almeida, Rossana Cavallo, Cristina Costa

**Affiliations:** 1 Microbiology and Virology Unit, University Hospital Città della Salute e della Scienza di Torino, Turin, Italy; 2 Department of Internal Medicine 4, Hospital de Santa Marta, Central Lisbon Hospital Centre, Lisbon, Portugal; 3 NOVA Medical School, Universidade Nova de Lisboa, Campo dos Mártires da Pátria 130, 1169-056 Lisbon, Portugal

**Keywords:** Bloodstream infection, CTX-M, Carbapenemase, Fast microbiology diagnostics, Enterobacterales, Antimicrobial stewardship

## Abstract

This study aims at presenting a reliable fast-track diagnostics for the detection of CTX-M ESBL- (CTX-M-p) and carbapenemase-producers (CA-p) directly from blood cultures (BCs) of patients with *Enterobacterales* (EB) bloodstream infections (BSIs) admitted in emergency and internal medicine departments and its contribution in estimation of in vitro antibiotic susceptibility. A fast-track workflow including MALDI-TOF species identification and two lateral flow immunochromatographic assays for the detection of CTX-M-p and CA-p directly from BCs was performed in parallel with conventional routine, and results were compared. A total of 236 BCs of patients suffering from EB BSI were included. Accuracy of the fast-track workflow ranged from 99.6 to 100%. Among *E. coli* isolates, CTX-M-p (20.5%) were susceptible to ceftolozane-tazobactam (C/T, 97%), ceftazidime-avibactam (CZA, 100%), and piperacillin-tazobactam (TZP, 84.8%), whereas CTX-M-and-main-carbapenemases-non-producer (CTX-M-CA-np, 79.5%) isolates were susceptible to all the antibiotics tested. Among *K. pneumoniae* isolates, CTX-M-p (23.3%) were poorly susceptible to TZP (40%) but widely susceptible to C/T (90%), CZA (100%), and amikacin (90%), whereas CTX-M-CA-np (55.8%) were also susceptible to cefepime. CA-p *K. pneumoniae* (20.9%) were susceptible to CZA (88.9%). All the species other than *E. coli* and *K. pneumoniae* were CTX-M-CA-np and were widely susceptible to the antibiotics tested except for isolates of the inducible and derepressed AmpC- or AmpC/ESBL-p species. Rapid identification of species and phenotype together with knowledge of local epidemiology may be crucial to determine the likelihood of deduction of in vitro antibiotic susceptibility on the same day of positive BC processing.

## Introduction

Extended-spectrum-β-lactamase (ESBL)- and carbapenemase-producing *Enterobacterales* (EB) bloodstream infections (BSIs) are a relevant clinical issue worldwide, especially given their association with limited therapeutic options, poor outcomes, and growing community-onset prevalence [1–5]. Since BSI patients are mainly managed in emergency and non-intensive wards [6, 7], rapid detection of ESBL- and carbapenemase-producing EB directly from positive blood cultures (BCs) in these settings is essential for early optimization of antibiotic therapy and improvement of clinical outcomes [8, 9]. Despite the complexity of the epidemiology, conjugative plasmids carrying CTX-M-type ESBL and KPC-, NDM-, VIM-, IMP-, and OXA-48-like genes were the main drivers of ESBL and carbapenemase dissemination in EB, respectively [1, 10, 11]. Recently, two lateral flow immunoassays (LFIAs), designed to detect CTX-M ESBL and the most widespread carbapenemases using specific monoclonal antibodies, have been optimally evaluated directly from positive EB BCs [12–14]. Despite fast microbiology workflows on selected cohorts of patients being highly encouraged [8, 15], there is limited evidence on their implementation and firepower in lab routine [16]. This study aims at presenting a reliable fast-track diagnostics for the detection of CTX-M ESBL- (CTX-M-p) and carbapenemase-producers (CA-p) from positive EB BCs of patients admitted in emergency and internal medicine departments. In the carbapenem-sparing era, rapid identification of species and phenotype together with knowledge of local epidemiology in areas of high ESBL- and carbapenemase-producing EB endemicity may be crucial to indicate the likelihood of deduction of in vitro antibiotic susceptibility and drive antibiotic management on the same day of positive BC processing, at least 24 h before than conventional testing results.

## Methods

### Conventional blood culture routine

Città della Salute e della Scienza di Torino in Turin, Italy, is a 2300-bed university hospital, with over 70,000 visits per year at the emergency department and about 300 beds in internal medicine wards. Our laboratory based at the Microbiology and Virology Unit is routinely open 7 days per week from 8 a.m. to 6 p.m. BACT/ALERT FA and FN Plus BC bottles (bioMérieux, Marcy l’Ètoile, France) are incubated in the BACT/ALERT Virtuo (bioMérieux, Marcy l’Ètoile, France) at various times each day. Positive BCs are subjected to Gram staining and subculture on appropriate solid medium when testing positive during routine laboratory working hours. Pathogen identification is performed on overnight subcultures using matrix-assisted laser desorption ionization-time of flight mass spectrometry (MALDI-TOF MS, Bruker DALTONIK GmbH, Bremen, Germany): a small amount of bacterial biomass is mixed with 1 μL of matrix solution and placed on the steel surface of the target plate to dry. The loaded target plate is inserted into the machine where it is then transported to the measuring chamber. Bacterial identification was considered reliable with a score of > 1.80. Antimicrobial susceptibility testing is performed on overnight subcultures using the Microscan WalkAway plus system according to the manufacturer’s instructions (Beckman Coulter, Brea, CA, USA). Antimicrobial susceptibilities are interpreted according to EUCAST breakpoints as updated in 2020 [17].

A disc-based phenotypic method evaluating inhibitory activity of clavulanate or cloxacillin on broad-spectrum-β-lactamases (total ESBL + AmpC Confirm kit, Rosco, Taastrup, Denmark) is used to identify ESBL- and AmpC-producers (AmpC-p) if cefotaxime and/or ceftazidime minimum inhibitory concentrations (MICs) were >1 mg/L. The Mastdiscs combi Carba plus disc system (Mast Group Ltd, Bootle, UK) is used to assess carbapenemase producers when meropenem MIC was > 0.125 mg/L. In case of phenotypic detection of ESBL- and/or carbapenemase-producers EB, NG-Test CTX-M MULTI and NG-Test Carba 5 assays are performed according to manufacturer’s instructions (NG Biotech, Guipry, France) to identify CTX-M ESBLs and the five most widespread carbapenemase families (OXA-48-like, KPC, NDM, IMP, and VIM), respectively.

Xpert Carba-R assay (Cepheid, Sunnyvale, CA) is also carried to detect the main carbapenem resistance genes when meropenem MIC was >0.125 mg/L and/or ceftazidime-avibactam (CZA) MIC ≥8 mg/L. All newly available identification and susceptibility results are promptly communicated to the clinicians and uploaded on to the laboratory information system.

### Study design

**Fig. 1:**
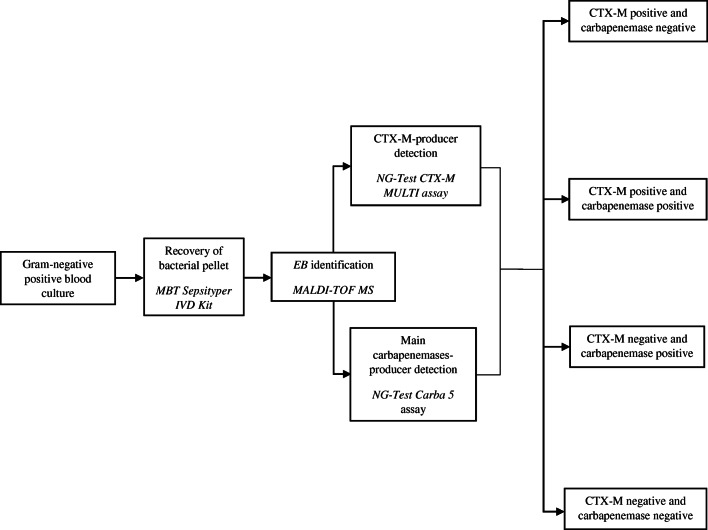
Fast-track workflow for positive *Enterobacterales* blood cultures of patients admitted in emergency and internal medicine departments

Over a 1-year period, conventional BC routine of patients admitted in emergency and internal medicine departments was performed in parallel with the fast-track workflow shown in Fig. 1. Fast-track workflow results were then compared with routine results to estimate the agreement between rapid and conventional phenotypic data. Subsequently, for each phenotypic EB group and species detected by the fast-track diagnostics, the matching likelihood of in vitro susceptibility according to conventional susceptibility testing results for some of the currently recommended antibiotics [18–22] was provided. Antibiotics included were piperacillin-tazobactam (TZP), ceftazidime (CAZ), cefepime (FEP), ceftolozane-tazobactam (C/T), CZA, and amikacin (AK). A multiplex real-time polymerase chain reaction assay specific for *bla*CTX-M-like genes (ESBL ELITe MGB Kit, ELITechGroup Molecular Diagnostics, Turin, Italy) to identify CTX-M ESBLs and Xpert Carba-R assay to detect the main carbapenem resistance genes were also carried out in case of discordance between fast-track diagnostics and conventional phenotypic results.

Time to detection of positivity (TtDP) was the time elapsed from when the BC bottle entered the incubation system to when it flagged as positive. Time to results (TtR) was defined as the time between the start of positive BC processing and availability of conventional and fast-track workflow results.

### Fast-track blood cultures workflow

Positive BCs of patients admitted in emergency and internal medicine departments, deemed representative of a single BSI event, and showing Gram-negative bacilli at Gram staining were subjected to recovery of bacterial pellet using the MBT Sepsityper IVD Kit (Bruker DALTONIK GmbH, Bremen, Germany). MALDI-TOF MS analysis of the bacterial pellets was then performed using the MALDI BioTyper system in accordance with the manufacturer’s instructions (Bruker DALTONIK GmbH, Bremen, Germany). Bacterial identification was considered reliable with a score of > 1.80. In case of EB identification, NG-Test CTX-M MULTI and NG-Test Carba 5 assays were performed adding ten drops of lysis buffer to the remaining bacterial pellet; after vortexing for 5 s, 100 μL of each suspension was added to the sample well of the respective test cassette, as previously described [12, 23]. According to LFIA results, all BCs included in the study were divided in three phenotypic group: (1) CTX-M-p (LFIAs tested CTX-M positive and main carbapenemases negative), (2) main-carbapenemases- or CTX-M-and-main-carbapenemases-producer (CA-p, LFIAs tested CTX-M negative and main carbapenemases positive or CTX-M positive and main carbapenemases positive), and (3.) CTX-M-and-main-carbapenemases-non-producer (CTX-M-CA-np, LFIAs tested CTX-M and main carbapenemases negative).

### Statistical analysis

Descriptive data are shown as absolute (*n*) and relative (%) frequencies for categorical data and as mean (range) for continuous variables. Accuracy, sensitivity, specificity, positive predictive value (PPV), and negative predictive value (NPV) of the fast-track workflow with 95% confidence interval (95% CI) were computed using the free software MedCalc website (http://medcalc.org/).

## Results

Over the study period a total of 855 BCs obtained from patients suffering from a Gram-negative BSI were processed. Among these, 261 (30.5%) belonged to patients admitted in emergency and internal medicine departments, of which 90.4% (*n*=236) were reliably identified from bacterial pellet as EB species by MALDI-TOF MS analysis and included in the assessment of the fast-track workflow. Overall, polymicrobial BCs involving EB species were 12 (4.9%). Of these, ten BCs were not considered for study purpose due to unreliable MALDI-TOF identification score. The EB species mainly observed were *Escherichia coli* (68.2%; *n*=161), *Klebsiella pneumoniae* (18.2%; *n*=43), *Enterobacter cloacae* (4.2%; *n*=10), and *Klebsiella aerogenes* (3%; *n*=7).

The mean TtDP was 12.3 h (range 2–68). The mean TtR for conventional and fast-track workflow were 38 h (range 27–78) and 42 min (range 35–55), respectively.

Accuracy, sensitivity, specificity, PPV, and NPV of the fast-track BCs workflow for the detection of CTX-M-p and CA-p were shown in Table 1. Fast-track and conventional BC workflows results were discordant in one case regarding a CZA resistant KPC-producing *K. pneumoniae* that was misclassified as CTX-M-CA-np by the fast-track workflow.

**Table 1:** Accuracy, sensitivity, specificity, positive predictive value, and negative predictive value of the fast-track blood cultures workflow for the detection of CTX-M ESBL- and carbapenemase-producers in patients admitted in emergency and internal medicine departments

Fast-track workflow results		Conventional phenotypic routine results		Accuracy, % (95% CI)	Sensitivity, % (95% CI)	Specificity, % (95% CI)	PPV, % (95% CI)	NPV, % (95% CI)
		Positive	Negative					
CTX-M-p	Positive	43	0	100 (98.5–100)	100 (91.8–100)	100 (98.1–100)	100	100
	Negative	0	193					
CA-p	Positive	8	0	99.6 (97.7–100)	88.9 (51.8–99.7)	100 (98.4–100)	100	99.6 (97.3–99.9)
	Negative	1	227					
CTX-M-CA-np	Positive	184	1	99.6 (97.7–100)	100 (98–100)	98.1 (89.7–100)	99.5 (96.4–99.9)	100
	Negative	0	51					

*Abbreviations*: *PPV* positive predictive value, *NPV* negative predictive value, *CTX-M-p* CTX-M-producer, *CA-p* main-carbapenemases-producer, *CTX-M-CA-np* CTX-M-and-main-carbapenemases-non-producer

Phenotypic characterization of EB species included in the study and likelihood of deduction of in vitro antibiotic susceptibility according to conventional culture-based results were reported in Table 2. Among *E. coli* isolates, 20.5% and 79.5% were CTX-M-p and CTX-M-CA-np, respectively. No CA-p *E. coli* were detected. CTX-M-p *E. coli* were in vitro susceptible to TZP (84.8%), C/T (97%), CZA (100%), and AK (75.8%). CTX-M-CA-np *E. coli* were largely in vitro susceptible to TZP (90.6%), CAZ (99.2%), FEP (100%), C/T (100%), CZA (100%), and AK (100%). Among AmpC/ESBL-np *E. coli* isolates, 9.5% (*n*=12) were resistant to TZP, whereas the only isolate resistant to CAZ was AmpC-p. Among *K. pneumoniae* isolates, 23.3%, 20.9%, and 55.8% were CTX-M-p, CA-p, and CTX-M-CA-np, respectively. CTX-M-p *K. pneumoniae* were in vitro susceptible to C/T (90%), CZA (100%), and AK (90%), whereas they showed susceptibility to TZP of 40%. All the CA-p *K. pneumoniae* isolates were pheno-genotypically characterized as KPC-producers, showing susceptibility to CZA and AK of 88.9% and 44.4%, respectively. Analysis of the KPC gene sequence of the only CZA-resistant CA-p *K. pneumoniae* isolate subsequently revealed to be a producer of KPC-31-carbapenemase harboring the mutation D179Y in the omega loop region of KPC-3 [23]. CTX-M-CA-np *K. pneumoniae* were in vitro susceptible to TZP (75%), CAZ (87.5%), FEP (100%), C/T (95.8%), CZA (100%), and AK (100%). Among AmpC/ESBL-np *K. pneumoniae*, 19.1% (*n*=4) were resistant to TZP, whereas among AmpC-p *K. pneumoniae*, 33% (*n*=1) and 66% (*n*=2) were susceptible to TZP and C/T, respectively. All the *E. cloacae* isolates were CTX-M-CA-np. They were in vitro susceptible to TZP (80%), CAZ (80%), FEP (80%), C/T (80%), CZA (100%), and AK (100%). All the AmpC/ESBL-p *E. cloacae* (*n*=2) were resistant to TZP, CAZ, FEP, and C/T. All the *K. aerogenes* isolates were CTX-M-CA-np. They were largely in vitro susceptible to FEP (85.7%), C/T (71.4%), CZA (100%), and AK (100%), whereas they showed poor susceptibility to TZP (28.6%) and CAZ (28.6%). Among AmpC-p *K. aerogenes* isolates, susceptibility to FEP and C/T were 100% (*n*=4) and 50% (*n*=2), respectively, whereas they were resistant to TZP and CAZ. The only AmpC/ESBL-p isolate showed resistance to TZP, CAZ and FEP, being susceptible to C/T. All the AmpC/ESBL-np isolates were susceptible to the antibiotics tested. All the *Klebsiella oxytoca*, *Proteus mirabilis*, *Citrobacter spp*, *Pantoea agglomerans*, *Morganella morganii*, and *Salmonella spp* isolates were CTX-M-CA-np, and they were susceptible to all the antibiotics tested. All the *Serratia marcenscens* isolates were CTX-M-CA-np, and they showed full susceptibility to FEP, C/T, and CZA. Susceptibility to TZP, CAZ, and AK was 50% since one of the isolates was phenotypically characterized as AmpC-p showing resistance to these antibiotics. The only *Hafnia alvei* isolate was classified as CTX-M-CA-np, being phenotypically characterized as AmpC-p, showing full susceptibility to TZP, FEP, CZA, AK, and resistance to CAZ, and C/T.

**Table 2:** Phenotypic characterization of *Enterobacterales* species included in the study and likelihood of deduction of in vitro antibiotic susceptibility according to conventional culture-based results

Species	Phenotypic results based on fast-track diagnostics					Likelihood of deduction of *in vitro* antibiotic susceptibility					
	CTX-M-p	CA-p	CTX-M-CA-np			TZP	CAZ	FEP	C/T	CZA	AK
			AmpC-p	AmpC/ESBL-p	AmpC/ESBL-np						
*E. coli* 68.2(161)	20.5 (33)	-	-	-	-	84.8 (28)	30.3 (10)	9.1 (3)	97 (32)	100 (33)	75.8 (25)
	-	-	0.6 (1)	-	78.9 (127)	90.6 (116)	99.2 (127)	100 (128)	100 (128)	100 (128)	100 (128)
*K. pneumoniae* 18.2(43)	23.3 (10)	-	-	-	-	40 (4)	0	0	90 (9)	100 (10)	90 (9)
	-	20.9 (9)	-	-	-	0	0	0	0	88.9 (8)	44.4 (4)
	-	-	7 (3)	-	48.8 (21)	75 (18)	87.5 (21)	100 (24)	95.8 (23)	100 (24)	100 (24)
*Other Enterobacterales 13.6(32)*											
*E. cloacae* 4.2(10)	-	-	-	20 (2)	80 (8)	80 (8)	80 (8)	80 (8)	80 (8)	100 (10)	100 (10)
*K. aerogenes* 3(7)	-	-	57.1 (4)	14.3 (1)	28.6 (2)	28.6 (2)	28.6 (2)	85.7 (6)	71.4 (5)	100 (7)	100 (7)
*K. oxytoca* 1.3(3)	-	-	-	-	100 (3)	100(3)	100 (3)	100 (3)	100 (3)	100 (3)	100 (3)
*P. mirabilis* 1.3(3)	-	-	-	-	100 (3)	100 (3)	100 (3)	100 (3)	100 (3)	100 (3)	100 (3)
*S. marcenscens* 0.9(2)	-	-	50 (1)	-	50 (1)	50 (1)	50 (1)	100 (2)	100 (2)	100 (2)	50 (1)
*Citrobacter spp* 0.9(2)	-	-	-	-	100 (2)	100 (2)	100 (2)	100 (2)	100 (2)	100 (2)	100 (2)
*P. agglomerans* 0.9(2)	-	-	-	-	100 (2)	100 (2)	100 (2)	100 (2)	100 (2)	100 (2)	100 (2)
*M. morganii* 0.4(1)	-	-	-	-	100 (1)	100 (1)	100 (1)	100 (1)	100 (1)	100 (1)	100 (1)
*Salmonella spp* 0.4(1)	-	-	-	-	100 (1)	100 (1)	100 (1)	100 (1)	100 (1)	100 (1)	100 (1)
*H. alvei* 0.4(1)	-	-	100 (1)	-	-	100 (1)	0	100 (1)	0	100 (1)	100 (1)

All data are shown as relative (%) and absolute (*n*) frequencies if not otherwise stated

*Abbreviations*: *CTX-M-p* CTX-M-producer, *CA-p* main-carbapenemases-producer, *CTX-M-CA-np* CTX-M-and-main-carbapenemases-non-producer, *AmpC-p* AmpC β-lactamase-producer, *AmpC/ESBL-p* AmpC/extended-spectrum β-lactamase-producer, *AmpC/ESBL-np* AmpC/extended-spectrum β-lactamase-non-producer, *TZP* piperacillin-tazobactam, *FEP* cefepime, *C/T* ceftolozane-tazobactam, *CZA* ceftazidime-avibactam, *CAZ* ceftazidime, *AK* amikacin

## Discussion

There was considerable agreement between the results provided by our fast-track workflow and conventional diagnostic methods. Despite the requirement for the later to confirm fast-track workflow results and to obtain a detailed pattern of drug activity, rapid tests for the detection of the more common resistance mechanisms in EB are an invaluable tool in setting timely antimicrobial stewardship interventions, thus potentially improving clinical outcomes, especially in BSI patients. Their implementation should prioritize patients at high risk for multidrug-resistant gram-negative bacteria infections to maximize cost-effectiveness [8]. In this 1-year study and considering disease prevalence, high PPV and NPV were found for our proposed fast-track workflow model.

The results provided were obtainable in less than 1 hour from the start of positive BC processing, which might favorably impact on antimicrobial stewardship interventions and be readily implemented into all laboratories with minimal training.

The fast-track BC workflow revealed to be particularly suited for the Italian and European epidemiology [1, 10], especially for cohorts of medical patients. In fact, EB were involved in almost all of BSI caused by gram-negative bacteria. *E. coli* and *K. pneumoniae* were the EB species mainly involved. They were found to be CTX-M-p in similar rates (20.5% and 23.3%, respectively), whereas carbapenemase production was observed only in *K. pneumoniae* isolates (20.9%). Similar epidemiological findings were found also by Fiori et al [16] that developed a timesaving and clinically relevant BC diagnostics based on the combined use of the MALDI BioTyper and a qualitative genotypic test to detect CTX-M-p and main CA-p in patients suffering from *E. coli* and *K. pneumoniae* BSI.

Concerning CA-p detection, since a CZA-resistant KPC-producing *K. pneumoniae* isolate was misclassified as CTX-M-CA-np by the fast-track diagnostics, a relevant issue emerged in this study [23]. In fact, KPC variants harboring mutations in omega loop, phenotypically characterized by impaired carbapenemase activity, restoration of carbapenems susceptibility, and expression of ESBL phenotype [24], can be undetected by LFIAs. Therefore, in patients with history of recent CZA treatment, a negative phenotypic result for KPC production should be confirmed by molecular assays.

Rapid detection of CTX-M-p, CA-p, or CTX-M-CA-np may also allow the prediction of multidrug resistance phenotypes and in vitro susceptibility based on local epidemiology of the EB species involved. Indeed, detection of CTX-M-p allows the early prediction of in vitro susceptibility to cephalosporins/beta-lactamase inhibitor combinations, such as C/T and CZA. TZP also showed a satisfactory in vitro activity against CTX-M-p *E. coli*, being its use considered according to BSI severity and source of infection [22]. Detection of KPC-producers suggests the probable efficacy of CZA, even though isolation of CZA-resistant KPC producing-EB could occur especially following CZA treatment. Detection of CTX-M-CA-np predicts in vitro susceptibility to CAZ among *E. coli* and *K. pneumoniae* isolates, but the presence of AmpC-p should be considered and switching to FEP always pondered. Of note, among CTX-M-CA-np *E. coli* and *K. pneumoniae*, 10.1% (*n*=15) that were classified as AmpC/ESBL-np showed in vitro TZP resistance and third-generation cephalosporin susceptibility. These strains were considered an emerging cause of BSI [25] and were the target of phenotypic and genotypic characterization [26] that did not fully elucidate the underlying resistance mechanism, even though they showed TZP activity on a murine model. Due to limited evidence on its generalizability to clinical practice, further studies are needed before considering TZP as a valid option against these strains in BSI patients. Among all the other than *E. coli* and *K. pneumoniae* EB, deduction of in vitro susceptibility should be based on EB species since the fast-track workflow classified all the isolates as CTX-M-CA-np. Therefore, empirical antibiotic therapy should consider the presence of inducible and derepressed AmpC- or AmpC/ESBL-p species (*Enterobacter* spp, *Citrobacter freundii*, *Serratia marcescens*, *Morganella morganii*, *Hafnia alvei*, and *Providencia* spp) [27] and avoid third generation cephalosporins [28] as well as TZP. These observations are limited due to the small sample size obtained in this study for these agents.

Strengths of this study include its pragmatic design and potential contribution to active programs of antimicrobial stewardship focused on selected cohorts of patients to maximize cost-effectiveness.

This study has some limitations. The fast-track BC workflow presented largely depends by a reliable direct MALDI-TOF identification and was designed according to the epidemiology of our hospital’s emergency and internal medicine departments and so it limits its generalizability to other settings. EB strains expressing carbapenemases other than those of the KPC type were not evaluated (i.e., *bla*VIM, *bla*IMP, *bla*OXA-48, *bla*NDM). Moreover, our fast-track BC workflow probably needs to be reviewed to maximize cost-effectiveness. Performing detection of CTX-M ESBL on both *E. coli* and *K. pneumoniae* species and detection of carbapenemases only on *K. pneumoniae* isolated in patients with history of recent hospitalization seems to be advisable. However, implementing fast-track diagnostics in lab routine means to be prompt to change it according to epidemiology and/or clinical reasons.

In conclusion, association of fast-track diagnostics for the detection of CTX-M-p and CA-p in BSI patients to conventional culture-based antimicrobial susceptibility testing in areas of high ESBL- and carbapenemase-producing EB endemicity may predict the likelihood of deduction of in vitro antibiotic susceptibility for each EB species on the same day of positive BC processing. Further studies are warranted to assess the impact of the implementation of fast-track BC workflow in antimicrobial stewardship programs on patient’s outcome and determining its cost effectiveness.
